# Perturbation-based dual task assessment in older adults with mild cognitive impairment

**DOI:** 10.3389/fresc.2024.1384582

**Published:** 2024-05-15

**Authors:** Lakshmi Kannan, Jessica Pitts, Tony Szturm, Rudri Purohit, Tanvi Bhatt

**Affiliations:** ^1^ Department of Physical Therapy, University of Illinois at Chicago, Chicago, IL, United States; ^2^ Department of Physical Therapy, University of Manitoba, Winnipeg, MB, Canada

**Keywords:** mild cognitive impairment, reactive balance, dual task, fall risk, cognition

## Abstract

**Background:**

Dual tasking (i.e., concurrent performance of motor and cognitive task) is significantly impaired in older adults with mild cognitive impairment (OAwMCI) compared to cognitively intact older adults (CIOA) and has been associated with increased fall risk. Dual task studies have primarily examined volitionally driven events, and the effects of mild cognitive impairment on reactive balance control (i.e., the ability to recover from unexpected balance threats) are unexplored. We examined the effect of cognitive tasks on reactive balance control in OAwMCI compared to CIOA.

**Methods:**

Adults >55 years were included and completed the Montreal Cognitive Assessment (MoCA) to categorize them as OAwMCI (MoCA: 18–24, *n* = 15) or CIOA (MoCA: ≥25, *n* = 15). Both OAwMCI [MoCA: 22.4 (2.2), 65.4 (6.1) years, 3 females] and CIOA [MoCA: 28.4 (1.3), 68.2 (5.5) years, 10 females] responded to large magnitude stance slip-like perturbations alone (single task) and while performing perceptual cognitive tasks targeting the visuomotor domain (target and tracking game). In these tasks, participants rotated their head horizontally to control a motion mouse and catch a falling target (target game) or track a moving object (track). Margin of stability (MOS) and fall outcome (harness load cell >30% body weight) were used to quantify reactive balance control. Cognitive performance was determined using performance error (target) and sum of errors (tracking). A 3 × 2 repeated measures ANOVA examined the effect of group and task on MOS, and generalized estimating equations (GEE) model was used to determine changes in fall outcome between groups and tasks. 2 × 2 repeated measures ANOVAs examined the effect of group and task on cognitive performance.

**Results:**

Compared to CIOA, OAwMCI exhibited significantly deteriorated MOS and greater number of falls during both single task and dual task (*p* < 0.05), and lower dual task tracking performance (*p* < 0.01). Compared to single task, both OAwMCI and CIOA exhibited significantly deteriorated perceptual cognitive performance during dual task (*p* < 0.05); however, no change in MOS or fall outcome between single task and dual task was observed.

**Conclusion:**

Cognitive impairment may diminish the ability to compensate and provide attentional resources demanded by sensory systems to integrate perturbation specific information, resulting in deteriorated ability to recover balance control among OAwMCI.

## 1 Introduction

Mild cognitive impairment is a prodromal state of dementia that manifests with subtle balance control and gait deficits which may affect activities of daily living (1) and contribute to the two folded increased risk of falls in this population (2, 3). Consequences of these falls lead to reduced physical functioning, dependency, and long-term disability (4, 5), ultimately increasing the likelihood to develop dementia or Alzheimer's disease (6, 7). A majority of falls occur during activities of daily living that involve performing a motor and cognitive task simultaneously (i.e., dual tasking) (8, 9). Therefore, understanding the biomechanical basis of balance control deficits under dual task conditions may inform the development of fall prevention strategies for this population.

Studies have used dual task methods to evaluate how individuals allocate cognitive/motor resources when simultaneously attending to two tasks (10, 11). If the attentional demands are greater than the capacity of an individual, dual tasking results in cognitive-motor interference, such that there is deterioration of performance on either or both tasks (12, 13). Previous studies have shown that older adults with mild cognitive impairment (OAwMCI) experience higher cognitive-motor interference than cognitively intact older adults (CIOA) during volitional balance control tasks, resulting in increased standing postural sway and reduced gait speed while performing a cognitive task (e.g., visual search, digit span recall, word recall) (14–17). However, activities of daily living do not only involve gait and working memory tasks, but also entail the ability to recover from unpredictable balance threats induced by the environment (i.e., reactive balance control) (18, 19). Understanding biomechanical factors contributing to cognitive-motor interference in OAwMCI compared to their healthy counterparts remains to be explored.

When an unexpected balance loss occurs, the CNS recruits feedback mechanisms to respond to the balance loss via compensatory strategies, which are modified online based on the perceived perturbation magnitude (20). In case of small magnitude perturbations, in-place ankle or hip strategies are recruited and as the magnitude becomes larger, a change in support strategy via stepping or grasping becomes necessary to recover postural stability (i.e., position and velocity of the COM relative to the displaced base of support) (18, 21). While such responses are triggered either by the short (spinal segmental) or long-loop (brain stem) reflexes, it is postulated that higher-cortical centers further relay the perturbation-specific sensory information (i.e., perturbation displacement, acceleration, and velocity) to optimize postural responses via the transcortical loop (22–24). Sensorimotor decline related to healthy aging can delay the ability to perceive and integrate sensorimotor information to initiate stepping, resulting in increased number of compensatory steps, delayed step initiation, and increased limb collisions in response to large intensity perturbations (25–27) compared to young adults (28). There is limited understanding whether a state of mild cognitive impairment could further reduce the ability to recover from unexpected external perturbations.

Our previous findings indicate that OAwMCI exhibit deteriorated reactive balance control compared to young adults and CIOA, including delayed step initiation time, reduced step length, and reduced postural stability when exposed to large magnitude perturbations (29). Further, OAwMCI were unable to modulate postural responses at higher perturbation intensities (29). These deficits are potentially attributable to the structural and functional cortical impairments observed in OAwMCI, as there is preliminary evidence that dual task reactive responses are worse among people with cortical lesions (e.g., stroke, Parkinson's disease, concussion injury) (30–36). However, these responses were observed in a controlled environment where the individual had nothing but the motor task (i.e., slip-like perturbations) to attend to. Real-life environments may incorporate the additional dynamics of perceptual cognitive demands, like standing and visually searching for cues, or standing and tracing items in the environment. Research has shown that OAwMCI experience a decline in visual processing capacity, visual search, and attention-related processing (37), which leads to difficulty processing moving visual scenes during standing, causing losses of balance (38). These scenarios may require more substantial attentional demands to recruit the appropriate motor strategies to recover from unexpected balance control threats, due to potential overlapping resources between reactive balance control and cognitive function. In line with this, our recent study in young adults showed that reactive postural stability in response to large magnitude perturbations was significantly lower while performing visuomotor games than during single task reactive balance (39).

There is no study to date that has examined reactive responses under dual task conditions challenging the perceptual cognitive function to understand the pattern of cognitive-motor interference in a real-life like environment in older adults with and without cognitive impairment. For this reason, this study primarily aims to determine the differences in single task and dual task reactive responses between CIOA and OAwMCI while performing two different perceptual cognitive tasks. We first hypothesize that OAwMCI will have reduced reactive balance control [indicated by reduced margin of stability (MOS), increased number of falls] compared to CIOA in both single and dual tasking. Secondly, we hypothesize that OAwMCI will show higher performance errors on cognitive tasks compared to CIOA during single and dual tasking. Lastly, we hypothesize that OAwMCI will demonstrate higher cognitive-motor interference than CIOA (i.e., greater reduction in performance in dual task vs. single task), due to difficulties allocating attentional resources in challenging conditions. Due to the constantly changing nature of cognitive demands in real-life tasks, examining the ability to recover from unexpected balance loss under perceptually challenging conditions could help understand the influence of cognitive function on reactive balance control.

## 2 Materials and methods

### 2.1 Participants

The study included older adults above the age of 55 years with (*n* = 15) or without (*n* = 15) cognitive impairment after obtaining a written informed consent. This study was approved by the University of Illinois at Chicago (UIC) institutional review board (#2021-0478). Participants were recruited by posting flyers at the UIC College of Applied Health Sciences building, nearby independent living senior centers, bus stops, train stations, and grocery stores.

### 2.2 Participants' eligibility

The Montreal Cognitive Assessment (MoCA) scale was used to classify older adults by their cognitive status (40). Older adults with a score greater than or equal to 25 out of 30 points were considered cognitively intact and those with a score ranging between 18 and 24 out of 30 points were considered as having mild cognitive impairment (40). Older adults with the presence of any neurological, cardiovascular, or musculoskeletal impairments as well as the inability to stand independently without an assistive device were excluded from the study, as such impairments may interfere with analysis and focus of the study. A heel bone density scan was performed for older adults using the Lunar Achilles Insight EXPII (General electric company, Milwaukee, Wisconsin, USA), and those with a T-score of less than −2.0 were classified as osteopenia or osteoporotic and were excluded (41, 42).

### 2.3 Sensory system testing

For assessing vision, participants were tested on the Snellen's chart and were allowed to wear corrective lenses (43). This test was done to ensure that participants could determine the objects that would appear on the screen from a specific distance while playing the visuospatial cognitive tasks (with their corrective lenses/glasses). Their visual acuity score must have been at least 20/40 with or without corrective lenses, otherwise, they were excluded. For assessing protective touch sensation of the feet, Semmes-Weinstein monofilament test was performed with 3.61 and 5.07 filaments. If participants were unable to feel 3.61 and 5.07 filaments, they were excluded (44).

### 2.4 Leg dominance test

Participants were asked to kick a cone kept in front of them to determine their leg dominance (45).

### 2.5 NIH cognitive toolbox tests

To assess differences in baseline cognitive performance between groups and confirm MCI classification, participants completed tests within the NIH cognitive toolbox, including the list sorting working memory test, pattern comparison processing speed test, flanker inhibitory control and attention test, and dimensional change card sort test (46).

#### 2.5.1 List sorting working memory test

Pictures of different foods and animals were displayed on a tablet screen, and participants were asked to list all of the displayed items in size order, listing first all of the foods and then the animals (47). Participants were scored based on the number of items correctly recalled, and scores are displayed as age-corrected percentile ranks within the NIH Toolbox's nationally representative sample.

#### 2.5.2 Pattern comparison processing speed test

Two pictures were displayed side-by-side on the tablet screen, and participants were asked to determine as quickly as possible whether the two images were identical (48). Participants were scored based on the number of items correctly answered in 85 s, and scores are again presented as age-corrected percentile ranks.

#### 2.5.3 Flanker inhibitory control task

Participants were presented with a row of multiple arrows, and asked to determine if the middle arrow was pointing in the same or opposite direction of the arrows flanking it (49). Participants were scored based on their combined accuracy and reaction time; computed scores range from 0 to 10, with 10 indicating the highest possible performance.

#### 2.5.4 Dimensional change card sort test

Participants were shown a series of pictures which varied in color (yellow or blue) and shape (ball or truck) (49). Participants were asked to match each picture to one of two target pictures either by color or shape. Participants were scored based on their combined accuracy and reaction time; computed scores range from 0 to 10, with 10 indicating the highest possible performance.

### 2.6 Clinical measures

The balance evaluation systems test (BESTest) was performed to determine differences in clinical balance scores between OAwMCI and CIOA (27). This test comprises of 36 items to assess performance on 6 balance control systems, namely biomechanical constraints, stability limits, anticipatory responses, postural responses, sensory orientation, and stability in gait.

### 2.7 Reactive balance control test in standing

A stance perturbation test was administered using the Active step (Simbex, Lebanon, NH) motorized treadmill, and the full-body kinematics were recorded via Qualisys using an eight-camera motion capture system (Qualisys, Gothenburg, Sweden) with a sampling rate of 120 Hz. A full body safety harness attached to a load cell via a pair of shock absorbing ropes further attached to a ceiling mounted metal track/beam secured the participants and prevented their knees from contacting the belt surface in case of a fall. A Helen Hayes marker set including 26 markers was placed bilaterally on bony landmarks to compute the center of mass (COM), and an additional marker was placed on the treadmill belt to identify the instant of perturbation onset (i.e., sudden treadmill belt acceleration). The load cell measured the amount of body weight exerted on the harness. Participants were asked to attain a comfortable stance position with their feet shoulder-width apart. They were instructed to execute a natural response to regain their balance by taking a step upon a sudden forward movement of the belt (slip-like perturbation). A familiarization trial was provided before the actual test and perturbation onset was unknown to participants. Perturbations for testing were induced by moving the belt forward with a displacement of 0.3 m for 0.38 s at 0.86 m/s with an acceleration of 21.5 m/s^2^. Four perturbations were induced within a given trial and duration between each perturbation was 6 s. Kinematic variables, such as margin of stability were computed using a custom-written algorithm in MATLAB version 2018b (The MathWorks Inc., Nactick, MA).

#### 2.7.1 Fall outcome

**Figure 1:**
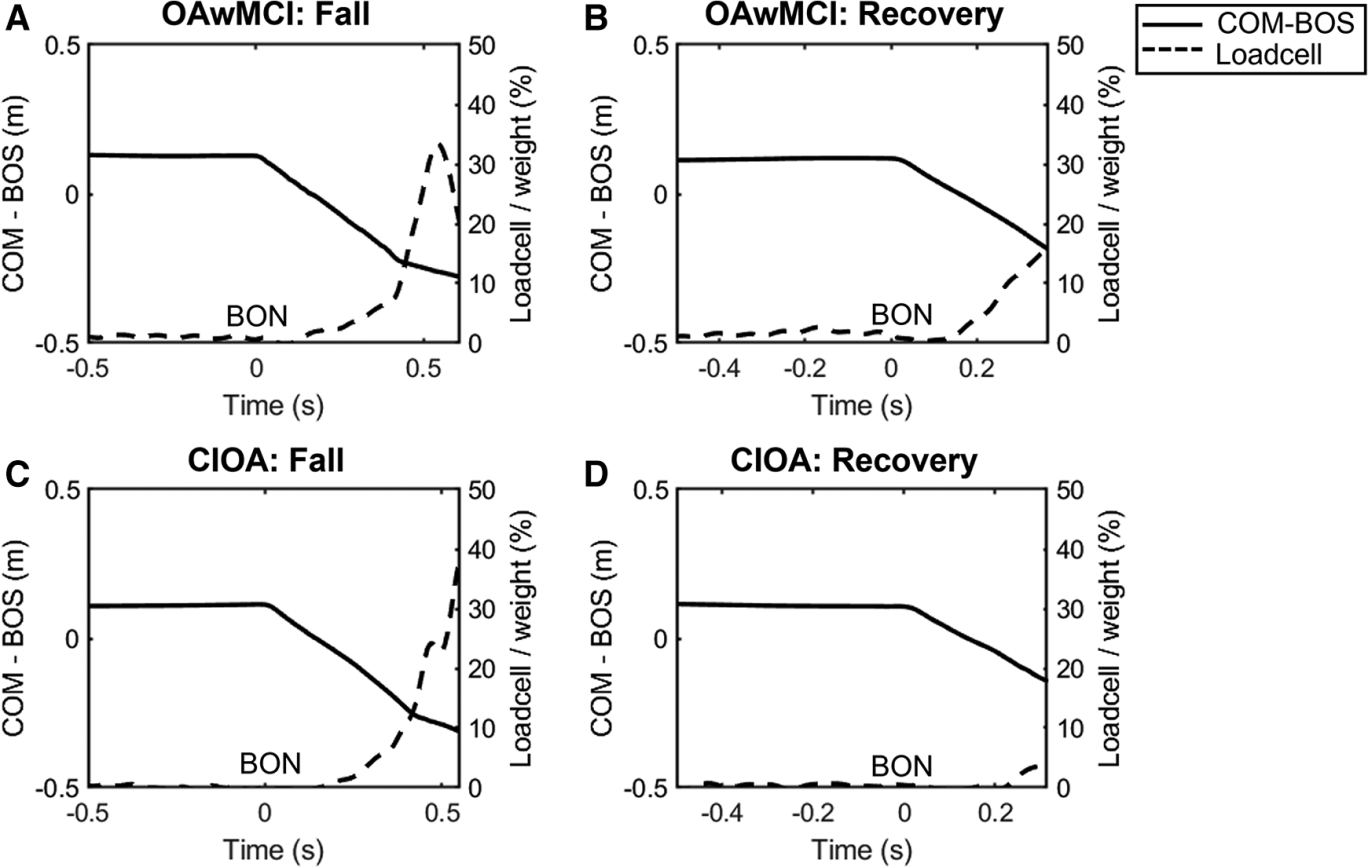
Representative plot from a single subject of the center of mass position relative to the base of support position (COM-BOS) (solid line) and percentage of body weight detected by the loadcell (dotted line) during a single-task perturbation exposure. Representative plots are presented during a fall and recovery for one older adult with mild cognitive impairment (OAwMCI) (**A,B**) and one cognitively intact older adult (CIOA) (**C,D**). A fall was identified if the peak load cell exceeded 30% of body weight. For all plots, belt onset occurs at 0 s, and the end of the plot signifies the instant of recovery touchdown. BON, belt onset; CIOA, cognitively intact older adult; COM-BOS, center of mass position relative to the base of support; OAwMCI, older adult with mild cognitive impairment.

A fall was identified if the peak load cell force during each perturbation exposure exceeded 30% of individual body weight (50). Figure 1 shows how falls were detected, displaying a representative tracing of the load cell (% body weight) during one perturbation exposure in the single task condition for a fall and recovery outcome. Additionally, this figure shows the relationship between the % body weight in the load cell and the relative COM position after perturbation onset. All falls were also visually verified using video recordings. There were four perturbations delivered during each trial in single and dual task. If participants fell on any of the perturbations during one trial, that trial was marked as a fall, otherwise as a recovery.

#### 2.7.2 Margin of stability

Margin of stability (MOS) was obtained post-perturbation onset to examine differences in reactive balance control between groups as well as differences between single and dual task. The margin of stability was computed by the following formula (51):

$$\mathrm{MOS}=\left(\mathrm{xCOM}+\frac{\mathrm{vCOM}}{\sqrt{\frac{g}{l}}}\right)/\mathrm{BO}S_{\mathrm{len}}$$

Here, the xCOM and vCOM are the center of mass position and velocity in the anteroposterior direction relative to the posterior edge of base of support (BOS). Gravitational acceleration is represented by *g* and leg length is denoted as *l* which was calculated using hip markers attached at the greater trochanter of the femur. The COM body kinematics were computed using a 13-segment rigid-body model with sex-dependent segmental inertial parameters. The BOS_len_ was the area beneath an individual encircled by the points of contact made by their foot or feet with the supporting surface in the anteroposterior direction. MOS values >1 indicate that the extrapolated COM exceeded the anterior boundary of the BOS, and negative values indicate that the COM exceeded the posterior boundary of the BOS. Specifically, for a slip perturbation, we expect all participants to experience a backward loss of balance; thus, higher MOS values indicate greater stability. Based on our pilot findings, it was observed that all older adults execute their first compensatory step within 0.4 s after belt onset, and a majority of the older adults with mild cognitive impairment directly leaned into the harness without initiating a step. We chose to analyze MOS at a fixed-time instance of 0.4 s post-belt onset to ensure that the movement of the treadmill belt (acceleration and position) was equivalent when comparing between trials. The treadmill belt contains different phases of movement during the perturbation delivery (e.g., accelerating/decelerating or moving constantly), which can affect the BOS dynamics and thus the outputted MOS. Further, we selected this time point to ensure that all participants had experienced a loss of balance when MOS was calculated.

### 2.8 Perceptual cognitive tasks

Visuomotor target and tracking games that challenged the visual and vestibular system were administered using a custom-built software, i.e., RTP (52). A motion sense mouse was placed over the participants head for the following games.

The target game required the participant to horizontally rotate their head (left and right) to interact with a paddle to catch a vertically dropped soccer ball while avoiding a distracter on the computer display for 45 s. Each soccer ball dropped at random locations at the top of the screen every 1.5 s with around 22–23 targets to catch within 45 s (52). Performance error was calculated by subtracting the actual distance they should have moved to catch the soccer ball from the maximum distance their head moved.

The tracking game required the participant to horizontally rotate their head left and right to follow a target which constantly moved horizontally on a screen for 45 s with a frequency of 0.4 Hz and fixed amplitude of 0.7 (i.e., 70% of monitor width/height) (52). Sum of errors is the number of overshoot errors and undershoot errors during the 45 s of the game.

These cognitive tasks were selected because they mimic head movements and visual tracking involved in common daily living scenarios (e.g., visually scanning the environment for postural threats, turning the head to check traffic while crossing the street). Both tasks required precise head-pointing movements and continual cognitive engagement, although required different strategies for optimal performance. The target game involved random appearance of objects on the display which could not be anticipated, thus requiring visual search to locate and catch the target object while simultaneously avoiding the distractor via cognitive inhibition (53). The tracking game involved following a target at a specific speed, where participants could anticipate and adapt to the target movement. This required moment by moment online visual feedback to determine the relative positions of the two objects, which involves smooth pursuit and the vestibulo-ocular reflex to maintain gaze stability (54). These perceptual cognitive tasks have been implemented to assess cognitive-motor interference during unperturbed walking (55, 56) and support surface perturbations in young adults (39). However, previous studies have not investigated the effect of similar tasks on reactive balance performance in CIOA or OAwMCI.

### 2.9 Dual task condition

Reactive balance control test was simultaneously performed with each of the cognitive tasks mentioned above in a randomized manner.

### 2.10 Statistical analysis

#### 2.10.1 Demographics and baseline clinical measures

Independent t-tests were conducted to determine the demographic differences between OAwMCI and CIOA (age, height, weight). Sex differences were determined using a chi-square test between the groups. Independent *t*-tests were conducted to determine differences in MoCA test, Balance evaluation systems test, and NIH cognitive toolbox assessments between OAwMCI and CIOA.

#### 2.10.2 Reactive balance control test

To determine dual task differences in reactive balance control, one 3 × 2 repeated measure ANOVA was conducted to examine the effect of group (OAwMCI vs. CIOA) and task (single vs. dual task) on the MOS (one for each of the different cognitive tasks) with age and gender as covariates. Post-hoc analyses with Bonferroni's correction were performed to resolve the main effects and interactions (Group × task). Generalized estimating equations (GEE) model was used to determine changes in fall incidence (binomial outcome) between groups and between single vs. dual task; a separate model was used to compare each dual task condition with the single task condition.

#### 2.10.3 Perceptual cognitive task

To determine dual task differences in cognitive task performance, two 2 × 2 repeated measure ANOVAs were conducted to compare the effect of group (OAwMCI vs. CIOA) and task (single vs. dual task) on performance error in visuomotor target game and sum of errors in the visuomotor tracking game. Post-hoc analyses with Bonferroni's correction were performed to resolve the main effects and interactions (Group × task).

## 3 Results

### 3.1 Demographics

The independent *t*-test between the OAwMCI and CIOA revealed that there were no significant differences in age [*p* = 0.18], height [*p* = 0.08], and weight [*p* = 0.98]. Furthermore, there were no sex differences [*X*^2^ (1, *N* = 33) = 1.48, *p* = 0.22] between the groups. Lastly, a significant difference in MoCA (*p* < 0.001) and balance evaluation systems test (BESTest) (*p* = 0.04) between the groups was observed. Additionally, there was a significant difference in cognitive scores on the list sorting test (*p* = 0.042), Flanker inhibitory control test (*p* < 0.001) and dimensional change card sort test (*p* < 0.001) between groups, which confirmed MCI status and deficits in cognitive functioning in the OAwMCI group. No significant values were detected with Levene's test and the data was considered to be normally distributed. Detailed characteristics are presented in Table 1.

**Table 1:** Demographics and clinical characteristics of older adults with mild cognitive impairment (OAwMCI) and cognitively intact older adults (CIOA).

	OAwMCI	CIOA	Between OAwMCI and CIOA *p* value
Age [Means (SD)]	65.41 (6.1)	68.19 (5.5)	0.186
Range in years	55–74	56–76	
Sex (M/F)	12/3	5/10	0.715
Height (cm) [Means (SD)]	171.27 (10.1)	165.61 (7.6)	0.081
Range in cm	149.86–182.88	152.4–182.88	
Weight (lbs) [Means (SD)]	160.88 (38.72)	161.19 (29.91)	0.98
Range in lbs	116–248	119–221	
BESTest Out of [Means (SD)]	92.25 (8.32)	97.69 (6.3)	0.04*
Range	83–107	86–106	
MoCA out of 30 [Means (SD)]	22.35 (2.2)	28.38 (1.31)	<0.001***
Range	18–25	26–30	
List sorting working memory test (Percentile) [Means (SD)]	24.70 (24.68)	52.08 (32.70)	0.042*
Range	2–86	9–99	
Pattern comparison test (Percentile) [Means (SD)]	34.66 (37.24)	58.25 (33.20)	0.132
Range	2–99.6	4–87	
Flanker task out of 10 [Means (SD)]	6.34 (1.17)	8.43 (0.64)	<0.001***
Range	5.00–8.22	7.37–9.79	
Dimensional change card sort test out of 10 [Means (SD)]	6.57 (1.02)	8.26 (0.81)	<0.001***
Range	4.78–8.34	7.35–9.98	

BESTest, balance evaluation systems test; MoCA, Montreal Cognitive Assessment.

*p < 0.05; ***p < 0.001.

### 3.2 Effect of dual tasking on reactive responses

#### 3.2.1 Fall outcome

The GEE model demonstrated no main effect of task or task by group interaction effect on fall outcome for either of the tasks (*p* > 0.05), indicating no difference in fall outcome between single task and dual task for either group. However, there was a significant main effect of group (OAwMCI vs. CIOA) on fall outcome for the visuomotor target game [*χ*^2^ (1) = 4.240, *p* = 0.039] and visuomotor tracking [*χ*^2^ (1) = 12.011, *p* < 0.001]. In single task, 73% of OAwMCI fell and 33% of CIOA fell. In the visuomotor target game, 73% of OAwMCI fell and 60% of CIOA fell. In visuomotor tracking, 87% of OAwMCI fell and 33% of CIOA fell.

#### 3.2.2 Margin of stability (MOS)

**Figure 2:**
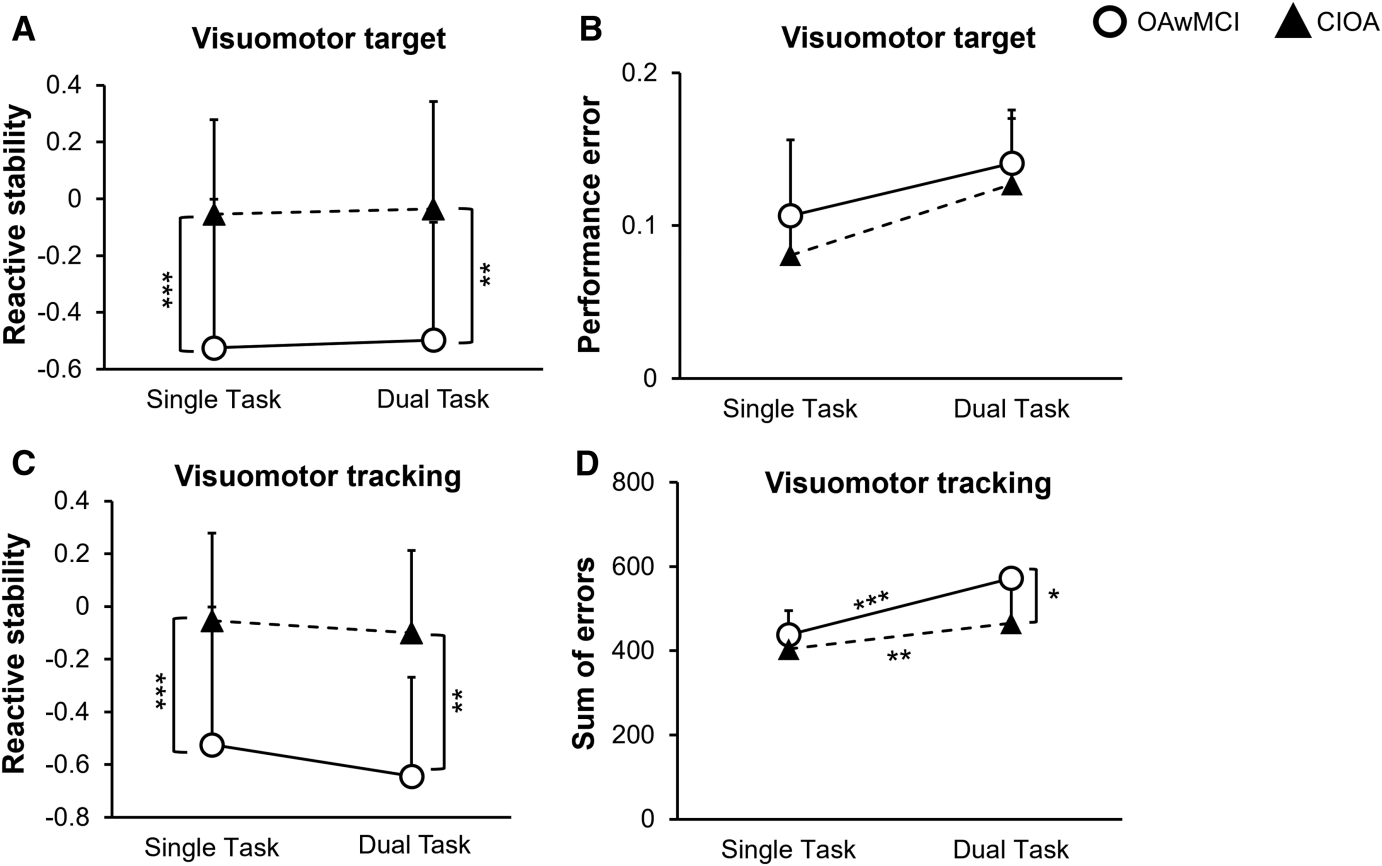
Differences in motor and cognitive performance between single task and dual task reactive balance in older adults with mild cognitive impairment (OAwMCI) and cognitively intact older adults (CIOA). Graphs represent motor performance (reactive stability) (**A**) and cognitive performance (performance error) (**B**) during the visuomotor target task, and motor performance (reactive stability) (**C**) and cognitive performance (sum of errors) (**D**) during the visuomotor tracking task, presented as means and standard deviations. *indicates *p* < 0.05; **indicates *p* < 0.01; ***indicates *p* < 0.001. CIOA, cognitively intact older adult; OAwMCI, older adult with mild cognitive impairment.

The 3 × 2 repeated measure ANOVA revealed a significant main effect of group (OAwMCI vs. CIOA) on the MOS [*F* (1,26) = 17.26, *p* < 0.001]. However, there was no main effect of task (single vs. either of the tasks) [*F* (2,52) = 1.92, *p* = 0.16] or task by group interaction effect [*F* (2,52) = 0.33, *p* = 0.72] or task by age [*F* (2,52) = 1.62, *p* = 0.21] or task by sex [*F* (2,52) = 1.15, *p* = 0.32] on MOS, indicating no difference in MOS between single task and dual task among the groups. Post-hoc analysis revealed that compared to CIOA, OAwMCI exhibited significantly lower margin of stability for visuomotor target game [*t* (28)=−3.18, *p* = 0.004] (Figure 2A), visuomotor tracking [*t* (28)=−4.31, *p* < 0.001] (Figure 2C), and single task [*t* (28)=−2.93, *p* = 0.007].

### 3.3 Cognitive tasks

#### 3.3.1 Visuomotor target game

The 2 × 2 repeated measure ANOVA revealed no main effect of task (stance vs. stance slips) [*F* (1,26) = 0.71, *p* = 0.41] or task × age [*F* (1,26) = 0.12, *p* = 0.73] or task × sex [*F* (1,26) = 1.11, *p* = 0.30] or task × group [*F* (1,26) = 0.06, *p* = 0.80] or main effect of group [*F* (1,26) = 1.11, *p* = 0.30] on performance error for the visuomotor target game (Figure 2B).

#### 3.3.2 Visuomotor tracking game

For the sum of errors in the visuomotor tracking, the 2 × 2 repeated measure ANOVA revealed a task by group interaction [*F* (1,26) = 4.8, *p* = 0.04], and main effect of group [*F* (1,26) = 5.26, *p* = 0.03]. However, there was no main effect of task (stance vs. stance slips) [*F* (1,26) = 1.14, *p* = 0.30] or task × age [*F* (1,26) = 0.26, *p* = 0.61] or task × sex [*F* (1,26) = 0.72, *p* = 0.40]. Post-hoc analysis revealed a significant difference between cognitive task performance during single task compared to dual tasking for both OAwMCI [*t* (14) = 4.71, *p* < 0.001] and CIOA [*t* (14) = 3.136, *p* = 0.007]. Additionally, OAwMCI exhibited significantly higher sum of errors during dual tasking compared to CIOA [*t* (28) = 2.52, *p* = 0.018] (Figure 2D).

## 4 Discussion

Our study primarily examined differences in reactive and cognitive responses between OAwMCI and CIOA during dual task reactive balance control (i.e., responding to slip perturbations while performing the perceptual cognitive tasks). We observed that OAwMCI exhibited significantly higher fall rate and lower reactive stability (measured via margin of stability) during dual tasking compared to CIOA, although there were no differences in reactive responses between single task and dual task slip conditions for both groups. Both groups had lower performance on the perceptual cognitive task in dual task compared to single task (measured via sum of errors), demonstrating motor-related cognitive interference (i.e., motor performance remains stable at the cost of paying less attention to cognitive task). However, OAwMCI demonstrated a greater reduction in cognitive performance than CIOA on the visuomotor tracking task, which possibly indicates that OAwMCI could be more affected by dual tasking than CIOA.

### 4.1 Reactive balance impairments in OAwMCI

Our results are in line with previous studies which have shown that reactive balance impairments are more pronounced in OAwMCI than CIOA (27, 29). CIOA already experience age-associated sensorimotor decline which affects reactive COM stability, although cognitive pathology related to MCI could further impair the ability to identify and interpret perturbation-specific information and subsequently disrupt sensory integration and/or motor execution (57–60). We confirmed using the NIH cognitive toolbox that OAwMCI had deficits in cognitive control, flexibility, and executive function, which may have contributed to delays in processing and responding to the postural threat. Specifically, OAwMCI may have significant impairment in recruiting and integrating visual, vestibular, and somatosensory information. The slip-like perturbations challenged all sensory (i.e., visual, vestibular, and somatosensory) systems, and the target and tracking game further loaded the visual and the vestibular system. Studies have shown that when any of the sensory systems are significantly impaired, it loads the other systems to compensate for the loss. This may demand resources from cognitive factors- like attention, visuospatial processing, working memory, executive function etc., for optimal performance, potentially delaying the triggering of compensatory responses and contributing to either a loss of balance or a fall when sufficient cognitive resources are not available. Even without the addition of the cognitive task, the OAwMCI group appeared unable to appropriately/effectively allocate resources to balance recovery compared to CIOA. While CIOA retained the capability to initiate compensatory stepping reactions on perturbation onset, many of OAwMCI were unable to initiate a compensatory step and directly experienced a fall.

### 4.2 Effect of dual tasking on reactive balance performance

We observed no effect of task on reactive balance (i.e., neither group had a difference in MOS between single and dual task conditions). This is contrasting to our previous study in young adults where MOS while performing visuomotor cognitive tasks was significantly lower compared to single task when exposed to forward support surface perturbations (39). The insignificant difference in motor performance between single and dual task in our study supports previous evidence that OAwMCI and CIOA may have utilized a “posture first” strategy under dual task conditions. Specifically, individuals may not have had sufficient attentional resources to divide between the ongoing cognitive and motor tasks, and subsequently prioritized motor performance at the cost of cognitive function, as exhibited by the reduction in cognitive performance during dual task compared to single task.

### 4.3 Effect of dual tasking on cognitive performance

We found that OAwMCI had lower performance than CIOA on the tracking game, although both groups had similar performance on the target game. This may have occurred due to differences in the nature of each task. While participants could not anticipate appearance of the objects on the display, participants could anticipate and adapt to the target movement. It is possible that neither group could adapt to the target task due to its unpredictability, whereas CIOA may have been better able to adapt to the movement of the tracking task, thus exhibiting lower performance error than OAwMCI. Recent studies show that age-associated vestibular dysfunction could contribute to increased cognitive decline in older adults (61–63). Additionally, vestibular dysfunction has been associated with hippocampal atrophy, which is significantly affected in OAwMCI and a biomarker of diagnosing early Alzheimer's disease (63). Such hippocampal atrophy affects the ability to process spatial information and memorize objects, thus affecting learning. Therefore, it could be that the combined age-associated decline with cognitive impairment could have further deteriorated the vestibular system to operate at its utmost functional capacity. This could have reduced the ability to adapt to the continuous movement, increasing the performance error among OAwMCI. Further, these results may indicate sharing of resources between cognitive function (specifically within the visuomotor domain) and reactive responses.

Although both groups had a decrease in cognitive performance during dual tasking, the difference in performance between single task and dual task on the tracking task was larger in OAwMCI than in CIOA. Thus, our findings suggest that cognitive-motor interference generated during the task affected OAwMCI greater than CIOA. The significant cognitive deficits in OAwMCI may impair their ability to allocate attention based on task demands, while CIOA appear better able to shift attentional resources in response to changing needs. It should also be considered that disruption of head movements caused by the sudden perturbation could have interfered with task performance, thus contributing to lower cognitive performance in dual task than single task. However, we believe that the physical effect of the perturbation on cognitive task performance was minimal, as both groups had an equal reduction in cognitive performance on the target task, even though OAwMCI had greater instability (i.e., were more physically affected by the perturbation). Thus, the deterioration in cognitive performance in dual task must at least partially be attributed to increased cognitive load, with the greater reduction in performance in OAwMCI on the tracking task attributable to higher cognitive-motor interference rather than instability. Future studies may consider examining the relationship between head position and reactive stability during similar tasks.

### 4.4 Limitations and future directions

This is the first study to examine the effect of dual tasking on reactive balance responses in OAwMCI and CIOA. The results should be interpreted in light of some limitations. It should be noted that our study focused on examining responses from biomechanical aspects and assessing the underlying neurophysiology may provide substantial evidence in understanding reactive responses in OAwMCI. Additionally, this study involved a sample size of 15 in each group with a focus on visuomotor and visuospatial domains of cognitive function. A higher sample size with a focus on other cognitive domains like working memory and attention may yield better effect size and offer an in-depth understanding of the effect of dual tasking on type of tasks involved during reactive response. Lastly, the addition of a young control group may provide better understanding reactive responses under attentional demanding conditions and future studies may consider adding a young control group.

### 4.5 Conclusion

The results showed that OAwMCI had significantly lower motor and cognitive performance during both single and dual tasking compared to CIOA; however, there was no effect of task (single task vs. dual task) on reactive balance performance. During dual task reactive balance, both OAwMCI and CIOA prioritized the motor response at the cost of cognitive function; however, the interference generated during the task affected OAwMCI more than CIOA. Cognitive pathology related to executive function, visuospatial, and working memory may interfere with the ability to provide attentional resources to integrate perturbation specific information. This may have delayed triggering reactive responses, thus resulting in deteriorated ability to recover balance control among OAwMCI.

## Data Availability

The raw data supporting the conclusions of this article will be made available by the authors, without undue reservation.

## References

[1] Jekel K Damian M Wattmo C Hausner L Bullock R Connelly PJ Mild cognitive impairment and deficits in instrumental activities of daily living: a systematic review. Alzheimer’s Res Ther. (2015) 7(1):1–20. 10.1186/s13195-015-0099-0 25815063 PMC4374414

[2] Tinetti ME Speechley M Ginter SF. Risk factors for falls among elderly persons living in the community. N Engl J Med. (1988) 319(26):1701–7. 10.1056/NEJM198812293192604 3205267

[3] van Dijk PT Meulenberg OG Van de Sande HJ Habbema JDF. Falls in dementia patients. Gerontologist. (1993) 33(2):200–4. 10.1093/geront/33.2.200 8468012

[4] Terroso M Rosa N Torres Marques A Simoes R. Physical consequences of falls in the elderly: a literature review from 1995 to 2010. Eur Rev Aging Phys Act. (2014) 11:51–9. 10.1007/s11556-013-0134-8

[5] Khow KS Visvanathan R. Falls in the aging population. Clin Geriatr Med. (2017) 33(3):357–68. 10.1016/j.cger.2017.03.002 28689568

[6] Jayakody O Blumen HM Breslin M Ayers E Lipton RB Verghese J Longitudinal associations between falls and future risk of cognitive decline, the motoric cognitive risk syndrome and dementia: the einstein ageing study. Age Ageing. (2022) 51(3):afac058. 10.1093/ageing/afac058 35290430 PMC8923158

[7] Livingston G Huntley J Sommerlad A Ames D Ballard C Banerjee S Dementia prevention, intervention, and care: 2020 report of the lancet commission. Lancet. (2020) 396(10248):413–46. 10.1016/S0140-6736(20)30367-6 32738937 PMC7392084

[8] Bergland A Pettersen AM Laake K. Falls reported among elderly Norwegians living at home. Physiother Res Int. (1998) 3(3):164–74. 10.1002/pri.138 9782519

[9] Woollacott M Shumway-Cook A. Attention and the control of posture and gait: a review of an emerging area of research. Gait Posture. (2002) 16(1):1–14. 10.1016/S0966-6362(01)00156-4 12127181

[10] Smith E Cusack T Cunningham C Blake C. The influence of a cognitive dual task on the gait parameters of healthy older adults: a systematic review and meta-analysis. J Aging Phys Act. (2017) 25(4):671–86. 10.1123/japa.2016-0265 28253049

[11] Plummer P Eskes G Wallace S Giuffrida C Fraas M Campbell G Cognitive-Motor interference during functional mobility after stroke: state of the science and implications for future research. Arch Phys Med Rehabil. (2013) 94(12):2565–74.e6. 10.1016/j.apmr.2013.08.002 23973751 PMC3842379

[12] Al-Yahya E Dawes H Smith L Dennis A Howells K Cockburn J. Cognitive motor interference while walking: a systematic review and meta-analysis. Neurosci Biobehav Rev. (2011) 35(3):715–28. 10.1016/j.neubiorev.2010.08.008 20833198

[13] Plummer-D'Amato P Brancato B Dantowitz M Birken S Bonke C Furey E. Effects of gait and cognitive task difficulty on cognitive-motor interference in aging. J Aging Res (2012) 2012:3–5. 10.1155/2012/583894 PMC3503314 23209905

[14] Kahya M Gouskova NA Lo OY Zhou J Cappon D Finnerty E Brain activity during dual-task standing in older adults. J Neuroeng Rehabil. (2022) 19(1):123. 10.1186/s12984-022-01095-3 36369027 PMC9652829

[15] Montero-Odasso MM Sarquis-Adamson Y Speechley M Borrie MJ Hachinski VC Wells J Association of dual-task gait with incident dementia in mild cognitive impairment: results from the gait and brain study. JAMA Neurol. (2017) 74(7):857–65. 10.1001/jamaneurol.2017.0643 28505243 PMC5710533

[16] Shimada H Makizako H Tsutsumimoto K Uemura K Anan Y Suzuki T. Cognitive function and gait speed under normal and dual-task walking among older adults with mild cognitive impairment. BMC Neurol. (2014) 14(1):1–8. 10.1186/1471-2377-14-1 24694100 PMC3994221

[17] Zhou J Manor B McCarten JR Wade MG Jor'dan AJ. The effects of cognitive impairment on the multi-scale dynamics of standing postural control during visual-search in older men. Front Aging Neurosci. (2023) 15:1068316. 10.3389/fnagi.2023.1068316 36761178 PMC9905142

[18] Maki BE McIlroy WE. The role of limb movements in maintaining upright stance: the “change-in-support” strategy. Phys Ther. (1997) 77(5):488–507. 10.1093/ptj/77.5.488 9149760

[19] Maki BE McIlroy WE. Control of rapid limb movements for balance recovery: age-related changes and implications for fall prevention. Age Ageing. (2006) 35(suppl_2):ii12–ii8. 10.1093/ageing/afl078 16926197

[20] Jacobs JV Horak F. Cortical control of postural responses. J Neural Transm. (2007) 114:1339–48. 10.1007/s00702-007-0657-0 17393068 PMC4382099

[21] Pai Y-C Bhatt TS. Repeated-slip training: an emerging paradigm for prevention of slip-related falls among older adults. Phys Ther. (2007) 87(11):1478–91. 10.2522/ptj.20060326 17712033 PMC2826275

[22] Burleigh A Horak F. Influence of instruction, prediction, and afferent sensory information on the postural organization of step initiation. J Neurophysiol. (1996) 75(4):1619–28. 10.1152/jn.1996.75.4.1619 8727400

[23] Timmann D Horak F. Perturbed step initiation in cerebellar subjects 1. Modifications of postural responses. Exp Brain Res. (1998) 119(1):73–84. 10.1007/s002210050321 9521538

[24] Timmann D Horak F. Perturbed step initiation in cerebellar subjects: 2. Modification of anticipatory postural adjustments. Exp Brain Res. (2001) 141(1):110–20. 10.1007/s002210100858 11685415

[25] Mansfield A Peters AL Liu BA Maki BE. Effect of a perturbation-based balance training program on compensatory stepping and grasping reactions in older adults: a randomized controlled trial. Phys Ther. (2010) 90(4):476–91. 10.2522/ptj.20090070 20167644

[26] Pai Y-C Rogers MW Patton J Cain TD Hanke TA. Static versus dynamic predictions of protective stepping following waist–pull perturbations in young and older adults. J Biomech. (1998) 31(12):1111–8. 10.1016/S0021-9290(98)00124-9 9882043

[27] Tangen GG Engedal K Bergland A Moger TA Mengshoel AM. Relationships between balance and cognition in patients with subjective cognitive impairment, mild cognitive impairment, and Alzheimer disease. Phys Ther. (2014) 94(8):1123–34. 10.2522/ptj.20130298 24764071

[28] Patel PJ Bhatt T. Does aging with a cortical lesion increase fall-risk: examining effect of age versus stroke on intensity modulation of reactive balance responses from slip-like perturbations. Neuroscience. (2016) 333:252–63. 10.1016/j.neuroscience.2016.06.044 27418344

[29] Kannan LN Bhatt TS. Perturbation-based balance assessment: examining reactive balance control in older adults with mild cognitive impairments. Physiol Int. (2021) 108(3):353–70. 10.1556/2060.2021.00181 34529584

[30] Dierijck JK. Multiple concussions and dual task paradigms: reactive postural perturbation management (Doctoral dissertation). University of British Columbia (2017). 10.14288/1.0356391

[31] Schinkel-Ivy A Huntley AH Inness EL Mansfield A. Timing of reactive stepping among individuals with sub-acute stroke: effects of ’single-task’and ‘dual-task’conditions. Heliyon. (2016) 2(10):9–10. 10.1016/j.heliyon.2016.e00186 PMC5103078 27861645

[32] Bhatt T Alqahtani S Patel P. Effect of dual-task on fall risk in chronic stroke survivors: examining reactive balance responses to forward perturbations in stance. Arch Phys Med Rehabil. (2016) 97(10):e20. 10.1016/j.apmr.2016.08.058

[33] Morenilla L Márquez G Sánchez JA Bello O López-Alonso V Fernández-Lago H Postural stability and cognitive performance of subjects with Parkinson’s disease during a dual-task in an upright stance. Front Psychol. (2020) 11:1256. 10.3389/fpsyg.2020.01256 32903649 PMC7438725

[34] Jacobs JV Nutt JG Carlson-Kuhta P Allen R Horak FB. Dual tasking during postural stepping responses increases falls but not freezing in people with Parkinson’s disease. Parkinsonism Relat Disord. (2014) 20(7):779–81. 10.1016/j.parkreldis.2014.04.001 24768615 PMC4058381

[35] Peterson DS Barajas JS Denney L Mehta SH. Backward protective stepping during dual-task scenarios in people with Parkinson’s disease: a pilot study. Neurorehabil Neural Repair. (2020) 34(8):702–10. 10.1177/1545968320935814 32633614

[36] Patel PJ Bhatt T. Attentional demands of perturbation evoked compensatory stepping responses: examining cognitive-motor interference to large magnitude forward perturbations. J Mot Behav. (2015) 47(3):201–10. 10.1080/00222895.2014.971700 25559427

[37] Bublak P Redel P Sorg C Kurz A Förstl H Müller HJ Staged decline of visual processing capacity in mild cognitive impairment and Alzheimer’s disease. Neurobiol Aging. (2011) 32(7):1219–30. 10.1016/j.neurobiolaging.2009.07.012 19713001

[38] Kucharik M Kosutzka Z Pucik J Hajduk M Saling M. Processing moving visual scenes during upright stance in elderly patients with mild cognitive impairment. PeerJ. (2020) 8:e10363. 10.7717/peerj.10363 33240666 PMC7680028

[39] Pitts J Kannan L Bhatt T. Cognitive task domain influences cognitive-motor interference during large-magnitude treadmill stance perturbations. Sensors. (2023) 23(18):7746. 10.3390/s23187746 37765803 PMC10534402

[40] Nasreddine ZS Phillips NA Bédirian V Charbonneau S Whitehead V Collin I The Montreal cognitive assessment, moca: a brief screening tool for mild cognitive impairment. J Am Geriatr Soc. (2005) 53(4):695–9. 10.1111/j.1532-5415.2005.53221.x 15817019

[41] Hans D Schott A Meunier P. Ultrasonic assessment of bone: a review. Eur J Med. (1993) 2(3):157–63. 8261057

[42] Zagzebski JA Rossman PJ Mesina C Mazess RB Madsen EL. Ultrasound transmission measurements through the os Calcis. Calcif Tissue Int. (1991) 49(2):107–11. 10.1007/BF02565130 1913288

[43] Holladay JT. Visual acuity measurements. J Cataract Refract Surg. (2004) 30(2):287–90. 10.1016/j.jcrs.2004.01.014 15030802

[44] Feng Y Schlösser FJ Sumpio BE. The semmes weinstein monofilament examination as a screening tool for diabetic peripheral neuropathy. J Vasc Surg. (2009) 50(3):675–82.e1. 10.1016/j.jvs.2009.05.017 19595541

[45] van Melick N Meddeler BM Hoogeboom TJ Nijhuis-van der Sanden MW van Cingel RE. How to determine leg dominance: the agreement between self-reported and observed performance in healthy adults. PLoS One. (2017) 12(12):e0189876. 10.1371/journal.pone.0189876 29287067 PMC5747428

[46] Weintraub S Dikmen SS Heaton RK Tulsky DS Zelazo PD Bauer PJ Cognition assessment using the nih toolbox. Neurology. (2013) 80(11_supplement_3):S54–64. 10.1212/WNL.0b013e3182872ded 23479546 PMC3662346

[47] Tulsky DS Carlozzi N Chiaravalloti ND Beaumont JL Kisala PA Mungas D Nih toolbox cognition battery (nihtb-cb): list sorting test to measure working memory. J Int Neuropsychol Soc. (2014) 20(6):599–610. 10.1017/S135561771400040X 24959983 PMC4426848

[48] Carlozzi NE Tulsky DS Chiaravalloti ND Beaumont JL Weintraub S Conway K Nih toolbox cognitive battery (nihtb-cb): the nihtb pattern comparison processing speed test. J Int Neuropsychol Soc. (2014) 20(6):630–41. 10.1017/S1355617714000319 24960594 PMC4424947

[49] Zelazo PD Anderson JE Richler J Wallner-Allen K Beaumont JL Weintraub S. Ii. Nih toolbox cognition battery (cb): measuring executive function and attention. Monogr Soc Res Child Dev. (2013) 78(4):16–33. 10.1111/mono.12032 23952200

[50] Yang F Pai Y-C. Automatic recognition of falls in gait-slip training: harness load cell based criteria. J Biomech. (2011) 44(12):2243–9. 10.1016/j.jbiomech.2011.05.039 21696744 PMC3390207

[51] Hof A Gazendam M Sinke W. The condition for dynamic stability. J Biomech. (2005) 38(1):1–8. 10.1016/j.jbiomech.2004.03.025 15519333

[52] Szturm T Sakhalkar V Boreskie S Marotta JJ Wu C Kanitkar A. Integrated testing of standing balance and cognition: test–retest reliability and construct validity. Gait Posture. (2015) 41(1):146–52. 10.1016/j.gaitpost.2014.09.023 25455701

[53] Nayak A Alhasani R Kanitkar A Szturm T. Dual-task training program for older adults: blending gait. Visuomotor Cogn Train Front Netw Physiol. (2021) 1:736232. 10.3389/fnetp.2021.736232 PMC10013153 36925571

[54] Szturm T Reimer KM Hochman J. Home-based computer gaming in vestibular rehabilitation of gaze and balance impairment. Games Health J. (2015) 4(3):211–20. 10.1089/g4h.2014.0093 26182066

[55] Nankar M Szturm T Marotta J Shay B Beauchet O Allali G. The interacting effects of treadmill walking and different types of visuospatial cognitive task: discriminating dual task and age effects. Arch Gerontol Geriatr. (2017) 73:50–9. 10.1016/j.archger.2017.07.013 28778023

[56] Ahmadi S Sepehri N Wu C Szturm T. Comparison of selected measures of gait stability derived from center of pressure displacement signal during single and dual-task treadmill walking. Med Eng Phys. (2019) 74:49–57. 10.1016/j.medengphy.2019.07.018 31623942

[57] Dickerson B Salat D Greve D Chua E Rand-Giovannetti E Rentz D Increased hippocampal activation in mild cognitive impairment compared to normal aging and ad. Neurology. (2005) 65(3):404–11. 10.1212/01.wnl.0000171450.97464.49 16087905 PMC4335677

[58] Leandri M Cammisuli S Cammarata S Baratto L Campbell J Simonini M Balance features in Alzheimer’s disease and amnestic mild cognitive impairment. J Alzheimer’s Dis. (2009) 16(1):113–20. 10.3233/JAD-2009-0928 19158427

[59] Wang L Goldstein FC Veledar E Levey AI Lah JJ Meltzer CC Alterations in cortical thickness and white matter integrity in mild cognitive impairment measured by whole-brain cortical thickness mapping and diffusion tensor imaging. Am J Neuroradiol. (2009) 30(5):893–9. 10.3174/ajnr.A1484 19279272 PMC2901819

[60] Zhang Y Schuff N Camacho M Chao LL Fletcher TP Yaffe K Mri markers for mild cognitive impairment: comparisons between white matter integrity and gray matter volume measurements. PLoS One. (2013) 8(6):e66367. 10.1371/journal.pone.0066367 23762488 PMC3675142

[61] Agrawal Y Smith PF Rosenberg PB. Vestibular impairment, cognitive decline and Alzheimer’s disease: balancing the evidence. Aging Ment Health. (2020) 24(5):705–8. 10.1080/13607863.2019.1566813 30691295 PMC6663651

[62] Bosmans J Jorissen C Gilles A Mertens G Engelborghs S Cras P Vestibular function in older adults with cognitive impairment: a systematic review. Ear Hear. (2021) 42(5):1119–26. 10.1097/AUD.0000000000001040 33974775

[63] Brandt T Schautzer F Hamilton DA Brüning R Markowitsch HJ Kalla R Vestibular loss causes hippocampal atrophy and impaired spatial memory in humans. Brain. (2005) 128(11):2732–41. 10.1093/brain/awh617 16141283

